# Considering Cultural and Contextual Resources in Loneliness and Health Studies

**DOI:** 10.1093/geroni/igaf122.1712

**Published:** 2025-12-31

**Authors:** David Camacho, Naomi Adjei, Mélanie Levasseur

## Abstract

Loneliness is a significant public health problem for older adults around the world. Cultural and contextual resources influence the experience of loneliness, and its impact on health outcomes. Yet, there is a dearth of literature that examines the experience of loneliness in non-westernized cultures, rural spaces, or under resourced settings. This symposium brings together gerontological scholars from counseling, occupational therapy, healthcare informatics, and social work to examine loneliness in older adults navigating French-Canadian, Latin American, and US contexts. Researchers will highlight unique cultural (e.g., linguistic, familism, gender) and contextual factors (e.g., rural, transitory housing, nascent geriatric healthcare infrastructure) that may contribute to the presence of loneliness and challenge intervention implementation. Researchers will discuss findings from projects that: 1) examine the relationship of loneliness and smoking behaviors in older adults living in transitory housing in the US; 2) explore physical health, social, and technology factors that individually and jointly enhance or assuage loneliness in older adults living in rural Indiana; 3) examine health factors that facilitate social participation, and barriers and facilitators to implementing socialization interventions to reduce isolation and loneliness in low-income French-Canadian older adults; and 4) synthesize findings on quantitative studies investigating the relationship of loneliness and cognitive health in more than 26,000 Latin American older adults. Together, these presentations acknowledge critical cultural and contextual challenges that influence the impact of loneliness on health. Further, presentations will inform loneliness prevention and treatment efforts and highlight unique strategies to reach and include underrepresented older adults.

